# Impact of the COVID-19 pandemic on pediatric out-of-hospital cardiac arrest outcomes in Japan

**DOI:** 10.1038/s41598-024-61650-x

**Published:** 2024-05-16

**Authors:** Ayako Chida-Nagai, Hiroki Sato, Hirokuni Yamazawa, Atsuhito Takeda, Naohiro Yonemoto, Yoshio Tahara, Takanori lkeda

**Affiliations:** 1 Department of Pediatrics, Hokkaido University Hospital, Kita 15, Nishi 7, Sapporo, Hokkaido 060-8638 Japan; 2 Department of Cardiology and Clinical Examination, Oita University, Yufu, Japan; 3 Advanced Trauma, Emergency and Critical Care Center, Oita University Hospital, Yufu, Japan; 4 Japanese Circulation Society with Resuscitation Science Study (JCS-ReSS) Group, Tokyo, Japan

**Keywords:** COVID-19, OHCA, Child, Bystander CPR, Medical research, Risk factors

## Abstract

This study investigates the impact of the COVID-19 pandemic on pediatric out-of-hospital cardiac arrest (OHCA) outcomes in Japan, aiming to address a critical research gap. Analyzing data from the All-Japan Utstein registry covering pediatric OHCA cases from 2018 to 2021, the study observed no significant changes in one-month survival, neurological outcomes, or overall performance when comparing the pre-pandemic (2018–2019) and pandemic (2020–2021) periods among 6765 cases. However, a notable reduction in pre-hospital return of spontaneous circulation (ROSC) during the pandemic (15.1–13.1%, *p* = .020) was identified. Bystander-initiated chest compressions and rescue breaths declined (71.1–65.8%, 22.3–13.0%, respectively; both *p* < .001), while bystander-initiated automated external defibrillator (AED) use increased (3.7–4.9%, *p* = .029). Multivariate logistic regression analyses identified factors associated with reduced pre-hospital ROSC during the pandemic. Post-pandemic, there was no noticeable change in the one-month survival rate. The lack of significant change in survival may be attributed to the negative effects of reduced chest compressions and ventilation being offset by the positive impact of widespread AED availability in Japan. These findings underscore the importance of innovative tools and systems for safe bystander cardiopulmonary resuscitation during a pandemic, providing insights to optimize pediatric OHCA care.

Due to the coronavirus disease 2019 (COVID-19) pandemic, there have been some reports about the outcome of out-of-hospital cardiac arrest (OHCA)^1–4^. While reports indicating a significant increase in OHCA cases due to the COVID-19 pandemic are relatively common, many of these studies focus on adult cases or all age groups, and research on the impact of COVID-19 on pediatric OHCA outcomes is notably scarce^5–8^.

The tendency to avoid contact with others during the COVID-19 pandemic has made rescue breaths seem more daunting for laypersons compared to chest compressions. In fact, there’s a report suggesting a decreased willingness to perform rescue breaths compared to chest compressions during the COVID-19 pandemic^9^.

Considering this, it's worrying that children, who are more likely to progress from respiratory arrest to cardiac arrest compared to adults, may have fewer opportunities to receive rescue breathing during the COVID-19 pandemic. This could potentially lead to a further increase in OHCA cases among children compared to adults.

On the other hand, in Japan, education on cardiopulmonary resuscitation (CPR) in schools and the dissemination of automated external defibrillators (AEDs) have progressed, which may mitigate the increase in OHCA cases due to the COVID-19 pandemic. In this study, we investigated the impact of the COVID-19 pandemic in Japan on pediatric OHCA cases.”

## Methods

Data pertaining to patients under 20 years of age with OHCA recorded from January 1, 2018, to December 31, 2021, were extracted from the All-Japan Utstein registry data maintained by the Fire and Disaster Management Agency of Japan^10^. The data covers all cases of pediatric OHCA in Japan. At the time this study was conducted, the legal age of adulthood in Japan is defined as 20 years old according to the Civil Code. Accordingly, this study targeted cases involving individuals under the age of 20.

This study adhered to the principles outlined in the Declaration of Helsinki. The registry data were acquired by a resuscitation science subcommittee of the Japanese Circulation Society following the approval of mandated government legal procedures, and the committee provided meticulously anonymized data. Our reporting approach aligns with the Strengthening the Reporting of Observational Studies in Epidemiology (STROBE) reporting guideline. This research is a retrospective study, and the need for informed consent was waived by the Hokkaido University Hospital Institutional Review Board since the records do not contain personally identifiable information. Based on the fact that the Ethical Guidelines for Life Science and Medical Research Involving Human Subjects issued by the Ministry of Health, Labour and Welfare stipulate that, in principle, anonymized processed information that has already been created does not need to be reviewed by an Ethical Review Board, the Hokkaido University Hospital Institutional Review Board did not conduct such review about this study.

To exclude the influence of factors such as improvements in medical care levels and changes in cardiovascular disease treatment guidelines and societal background in Japan, we deemed it appropriate to compare the two years immediately preceding the COVID-19 pandemic, namely 2018–2019, with the subsequent two years. The number of pediatric OHCA cases in Japan during these years were as follows: 2018 (1738 cases), 2019 (1786 cases), 2020 (1658 cases), and 2021 (1584 cases). We excluded patients without information on the primary outcome measure and secondary outcomes from the analysis. Consequently, one patient was excluded, and the final analysis was conducted on 6765 cases.

The primary outcome measure was the one-month survival, with secondary outcomes including prehospital return of spontaneous circulation (ROSC), neurologically outcomes based on Cerebral Performance Categories (CPC), and overall outcomes based on Overall Performance Categories (OPC).

Group comparisons were conducted using the chi-square test for categorical variables and the Wilcoxon Mann–Whitney test for continuous variables. Multivariate logistic regression analyses were performed for patients before (2018–2019) and after (2020–2021) the onset of the COVID-19 pandemic. Based on previous reports^11,12^,” witnessed cardiac arrest”, “bystander-initiated chest compressions”, “bystander-initiated rescue breaths”, “bystander AED use”, “ventricular fibrillation (VF) or pulseless ventricular tachycardia (VT)”, and “Time from call ambulance to hospital” was selected as the evaluation item for the logistic regression analyses. All tests were two-sided, and a *p*-value < 0.05 indicated statistical significance.

## Results

Between 2018 and 2021, data from 6,765 pediatric OHCA patients were recorded and analyzed. When comparing pre-COVID-19 (2018–2019) and during-COVID-19 (2020–2021) periods, there were no significant changes observed in the one-month survival proportion, CPC, or OPC. However, the proportion of prehospital ROSC significantly decreased after the onset of the COVID-19 pandemic (Table 1). The causes of cardiac arrest and the initial electrocardiographic waveform at the time of emergency medical service contact were examined. No significant differences were observed between pre-COVID-19 period group and during-COVID-19 period group. The details are presented in Table 1.

**Table 1:** Characteristics of the pediatric OHCA patients (N = 6765).

Variables	Total (%) (N = 6765)	Pre-COVID-19 2018–2019 (%) (N = 3524)	During-COVID-19 2020–2021 (%) (N = 3241)	*p* value (2018–2019 vs 2020–2021)
Male	4122 (80.9)	2160 (61.3)	1962 (60.5)	.524
Witnessed cardiac arrest	2433 (36.0)	1290 (36.6)	1143 (35.3)	.252
Bystander-initiated chest compressions	4062 (68.4)	2111 (71.1)	1951 (65.8)	** < .001**
Bystander-initiated rescue breaths	897 (17.6)	548 (22.3)	349 (13.0)	** < .001**
Bystander-AED use	215 (4.3)	85 (3.7)	130 (4.9)	**.029**
Verbal guidance from a dispatcher	3915 (61.2)	2106 (64.1)	1809 (58.1)	** < .001**
Presence of a physician in the ambulance	585 (8.6)	305 (8.7)	280 (8.6)	.982
Time from call ambulance to hospital (minutes)	31 ± 15	30 ± 15	32 ± 16	** < .001**
The cause of cardiac arrest				
Cardiac etiology	2108 (31.2)	1090 (30.9)	1018 (31.4)	.671
Non-cardiac etiology	4657 (68.8)	2434 (69.1)	2223 (68.6)	
The initial ECG waveform at the time of emergency medical services contact				.105
VF	209 (3.1)	109 (3.1)	100 (3.1)	
pulseless VT	8 (0.1)	8 (0.2)	0	
PEA	1052 (15.6)	551 (15.6)	501 (15.5)	
Asystole	4648 (68.7)	2409 (68.4)	2239 (69.1)	
Others	848 (12.5)	447 (12.7)	401 (12.4)	
Prehospital ROSC	959 (14.2)	533 (15.1)	426 (13.1)	**.020**
One-month survival	1265 (18.7)	682 (19.3)	583 (18.0)	.150
CPC				.201
Good function	669 (9.9)	373 (11)	296 (9.1)	
Moderate disability	75 (1.1)	39 (1.1)	36 (1.1)	
Severe disability	142 (2.1)	81 (2.3)	61 (1.9)	
Coma or vegetative state	341 (5.0)	171 (4.9)	170 (5.3)	
Death or brain death	5538 (81.9)	2860 (81.2)	2678 (82.6)	
OPC				.242
Good function	671 (9.9)	374 (11)	297 (9.2)	
Moderate disability	72 (1.1)	39 (1.1)	33 (1.0)	
Severe disability	147 (2.1)	82 (2.3)	65 (2.0)	
Coma or vegetative state	337 (5.0)	169 (4.8)	168 (5.2)	
Death or brain death	5538 (81.9)	2860 (81.2)	2678 (82.6)	

*OHCA* out-of-hospital cardiac arrest; *ROSC* return of spontaneous circulation; *CPC* Cerebral Performance Categories; *OPC* Overall Performance Categories; *ECG* electrocardiogram; *VF* ventricular fibrillization; *VT* ventricular tachycardia; *PEA* pulseless electrical activity; *AED* automated external defibrillator. Bold values denote statistical significance at *p* < .05.

Furthermore, following the COVID-19 pandemic, the proportions of bystander-initiated chest compressions and rescue breaths by the bystander significantly decreased. On the other hand, the proportion of automated external defibrillator (AED) use by the general public significantly increased. To explore the factors contributing to the decreased proportion of prehospital ROSC during the pandemic, a multivariate logistic regression analysis was conducted. The results indicated that all of the following were significantly associated: ”witnessed cardiac arrest”, “bystander-initiated chest compressions”, “bystander-initiated rescue breaths”, “bystander AED use”, “VF or pulseless VT”, and “Time from call ambulance to hospital” (Table 2). The results of the multiple imputation were consistent with the previously mentioned findings.

**Table 2:** Multivariate logistic regression analysis for prehospital ROSC in all pediatric OHCA patients (N = 4970).

	OR	95% CI	*p* value
Witnessed cardiac arrest	5.39	4.42–6.58	** < .001**
Bystander-initiated chest compressions	3.06	2.43–3.84	** < .001**
Bystander-initiated rescue breaths	1.75	1.39–2.21	** < .001**
Bystander AED use	3.62	2.56–5.12	** < .001**
VF or pulseless VT	3.13	2.12–4.62	** < .001**
Time from call ambulance to hospital (minutes)	1.02	1.02–1.03	** < .001**

*OR* odds ratio; *CI* confidence interval; *AED* automated external defibrillator. *VF* ventricular fibrillization; *VT* ventricular tachycardia.

Bold values denote statistical significance at *p* < .05.

## Discussion

Our study, for the first time globally, has uncovered a significant reduction in the prehospital ROSC among pediatric OHCA patients following the COVID-19 pandemic, without any observed changes in the one-month survival rate. Additionally, our findings highlight the crucial associations between ”witnessed cardiac arrest”, “bystander-initiated chest compressions”, “bystander-initiated rescue breaths”, “bystander AED use”, “VF or pulseless VT”, and “Time from call ambulance to hospital” with the prehospital ROSC proportions in pediatric OHCA patients.

A noteworthy observation is the apparent decrease in the proportions of "bystander-initiated chest compression” and “bystander-initiated rescue breath" for pediatric OHCA patients after the onset of the COVID-19 pandemic. These declines are believed to be influenced by a general tendency among the public to avoid contact with others due to fear of COVID-19 infection^13,14^. Particularly, the decrease in rescue breaths may reflect the psychological concern of rescuers regarding the risk of exposure to others' bodily fluids during the COVID-19 pandemic. If the pandemic duration in Japan were more prolonged, or if there were a higher number of COVID-19-related fatalities, the one-month survival rates might have declined further due to increased avoidance of interpersonal contact. Further analysis is warranted to explore these possibilities.

In our study, deterioration was observed only in the ROSC after the onset of the COVID-19 pandemic, with no changes noted in the one-month survival rate and neurological outcomes. Explaining all the reasons for this phenomenon is challenging, however, we focus on that the proportion of "bystander AED use" for pediatric OHCA patients has increased after the COVID-19 pandemic. This can be attributed to the yearly increase in the number of AED installations in Japan and the advancements in CPR education^15,16^. As of 2019, Japan boasted a world-leading AED installation rate of 45.4 units per 10,000 people^17^. The authors believe that the rapid proliferation of AEDs in Japan may have contributed to some extent in preventing the deterioration of one-month survival rates and neurological outcomes. Moreover, taking into account the meta-analysis indicating an increase in OHCA at home due to COVID-19^18^, it becomes apparent that if the management of OHCA at home improves, there is potential for further improvement in OHCA survival rates. Hence, effective consideration of not only the increase in the number of AEDs but also their placement locations is essential.

In this study, the reason for the decrease in Dispatcher CPR instruction during the COVID-19 pandemic is unclear. Reports from Japan suggest both an increase in Dispatcher CPR instruction during the COVID-19 pandemic^19–21^ and no significant change^22^. While the importance of Dispatcher CPR instruction has been increasingly recognized over the years, it is possible that the strain on the emergency medical system that occurred in Japan during the COVID-19 pandemic may have contributed to the decrease in Dispatcher CPR instruction^23^. Further investigation is needed on this point.

There is a previous study that examined the impact of the COVID-19 pandemic on pediatric OHCA in Japan^24^. This study reported that the COVID-19 outbreak did not affect the ROSC or survival rates of pediatric OHCA, which differs from our findings. The previous study evaluated two groups of pediatric OHCA cases from 2015 to 2019 and 2020. We are concerned about the impact of the increasing number of AED installations and the spread of CPR education on the study results, and thus, we evaluated two groups from just before the COVID-19 outbreak in 2018–2019 and during the outbreak in 2020–2021. This difference in evaluation periods between the previous study and ours may contribute to the discrepancy in results.

A limitation of this study is the lack of information on the COVID-19 infection status of the targeted patients in the All-Japan Utstein registry data. Besides, in this registry, the evaluation of the attribute of bystander and the location of out-of-hospital cardiac arrests is inadequate. Since differences in the location of occurrence may affect survival rates, further investigation is necessary. Nevertheless, our findings in this study underscore the importance of reinforcing bystander CPR efforts and AED usage even during the COVID-19 pandemic, emphasizing that these interventions play a crucial role in improving outcomes for pediatric OHCA patients. There is a call for the development of tools and systems that enable bystanders to perform CPR without hesitation while protecting themselves from viral infections.

In conclusion, despite the formidable challenges posed by the COVID-19 pandemic, our study emphasizes the paramount importance of reinforcing bystander CPR efforts and utilizing AEDs in improving outcomes for pediatric OHCA cases. Based on the findings of this study, to enhance outcomes for pediatric OHCA patients, further promotion and awareness of CPR and AED utilization are needed. Additionally, promoting safe bystander CPR during a pandemic requires the development and widespread distribution of pediatric pocket resuscitation masks designed to protect bystanders from infectious diseases, along with the broad implementation of rescue gloves, which are deemed essential.

## Data Availability

The datasets used and/or analysed during the current study available from the corresponding author on reasonable request.

## Abbreviations

AED: Automated external defibrillator
COVID-19: Coronavirus disease 2019
CPR: Cardiopulmonary resuscitation
CPC: Cerebral performance categories
OHCA: Out-of-hospital cardiac arrest
OPC: Overall performance categories
ROSC: Return of spontaneous circulation
VF: Ventricular fibrillation
VT: Ventricular tachycardia

## References

[1] Ahn JY, Ryoo HW, Cho JW, Kim JH, Lee SH, Jang TC (2021). Impact of the COVID-19 outbreak on adult out-of-hospital cardiac arrest outcomes in Daegu, South Korea: An observational study. Clin. Exp. Emerg. Med..

[2] Chavez S, Huebinger R, Chan HK (2022). The impact of COVID-19 on incidence and outcomes from out-of-hospital cardiac arrest (OHCA) in Texas. Am. J. Emerg. Med..

[3] Kennedy C, Alqudah Z, Stub D, Anderson D, Nehme Z (2023). The effect of the COVID-19 pandemic on the incidence and survival outcomes of EMS-witnessed out-of-hospital cardiac arrest. Resuscitation.

[4] Coute RA, Nathanson BH, Kurz MC, Mader TJ (2022). Estimating the impact of the COVID-19 pandemic on out-of-hospital cardiac arrest burden of disease in the United States. J. Am. Coll. Emerg. Physicians Open.

[5] Lai PH, Lancet EA, Weiden MD (2020). Characteristics associated with out-of-hospital cardiac arrests and resuscitations during the novel coronavirus disease 2019 pandemic in New York City. JAMA Cardiol..

[6] Marijon E, Karam N, Jost D (2020). Out-of-hospital cardiac arrest during the COVID-19 pandemic in Paris, France: A population-based, observational study. Lancet Public health.

[7] Fothergill RT, Smith AL, Wrigley F, Perkins GD (2021). Out-of-hospital cardiac arrest in London during the COVID-19 pandemic. Resuscitation plus.

[8] Glober NK, Supples M, Faris G (2021). Out-of-hospital cardiac arrest volumes and characteristics during the COVID-19 pandemic. Am. J. Emerg. Med..

[9] Grunau B, Bal J, Scheuermeyer F (2020). Bystanders are less willing to resuscitate out-of-hospital cardiac arrest victims during the COVID-19 pandemic. Resusc. Plus.

[10] Kitamura T, Iwami T, Kawamura T, Nagao K, Tanaka H, Hiraide A (2010). Nationwide public-access defibrillation in Japan. New Engl. J. Med..

[11] Myat A, Song KJ, Rea T (2018). Out-of-hospital cardiac arrest: Current concepts. Lancet.

[12] Van de Voorde P, Turner NM, Djakow J (2021). European resuscitation council guidelines 2021: Paediatric life support. Resuscitation.

[13] Nakayachi K, Ozaki T, Shibata Y, Yokoi R (2020). Why Do Japanese people use masks against COVID-19, even though masks are unlikely to offer protection from infection?. Front. Psychol..

[14] Santomauro DF, Herrera AMM, Shadid J, Zheng P, Ashbaugh C, Pigott DM, Abbafati C, Adolph C, Amlag JO, Aravkin AY, Bang-Jensen BL (2021). Global prevalence and burden of depressive and anxiety disorders in 204 countries and territories in 2020 due to the COVID-19 pandemic. Lancet.

[15] Kiyohara K, Sado J, Kitamura T (2019). Public-access automated external defibrillation and bystander-initiated cardiopulmonary resuscitation in schools: A nationwide investigation in Japan. EP Europace.

[16] Nakashima T, Noguchi T, Tahara Y (2019). Public-access defibrillation and neurological outcomes in patients with out-of-hospital cardiac arrest in Japan: A population-based cohort study. Lancet.

[17] Ruan Y, Sun G, Li C (2021). Accessibility of automatic external defibrillators and survival rate of people with out-of-hospital cardiac arrest: A systematic review of real-world studies. Resuscitation.

[18] Masuda Y, Teoh SE, Yeo JW (2022). Variation in community and ambulance care processes for out-of-hospital cardiac arrest during the COVID-19 pandemic: A systematic review and meta-analysis. Sci. Rep..

[19] Hosomi S, Zha L, Kiyohara K (2022). Impact of the COVID-19 pandemic on out-of-hospital cardiac arrest outcomes in older adults in Japan. Resusc. Plus.

[20] Ushimoto T, Yao S, Nunokawa C, Murasaka K, Inaba H (2023). Association between the COVID-19 pandemic in 2020 and out-of-hospital cardiac arrest outcomes and bystander resuscitation efforts for working-age individuals in Japan: A nationwide observational and epidemiological analysis. Emerg. Med. J..

[21] Tanaka Y, Okumura K, Yao S, Okajima M, Inaba H (2023). Impact of the COVID-19 pandemic on prehospital characteristics and outcomes of out-of-hospital cardiac arrest among the elderly in Japan: A nationwide study. Resusc. Plus.

[22] Nishiyama C, Kiyohara K, Iwami T (2021). Influence of COVID-19 pandemic on bystander interventions, emergency medical service activities, and patient outcomes in out-of-hospital cardiac arrest in Osaka City, Japan. Resusc. Plus.

[23] Kokudo N, Sugiyama H (2021). Hospital capacity during the COVID-19 pandemic. Global Health Med..

[24] Zha L, Hosomi S, Kiyohara K, Sobue T, Kitamura T (2022). Association of the COVID-19 pandemic with prehospital characteristics and outcomes of pediatric patients with out-of-hospital cardiac arrest in Japan, 2005–2020. JAMA Netw. Open.

